# The Influence of Sintering Temperature on the Pore Structure of an Alkali-Activated Kaolin-Based Geopolymer Ceramic

**DOI:** 10.3390/ma15072667

**Published:** 2022-04-05

**Authors:** Mohd Izrul Izwan Ramli, Mohd Arif Anuar Mohd Salleh, Mohd Mustafa Al Bakri Abdullah, Ikmal Hakem Aziz, Tan Chi Ying, Noor Fifinatasha Shahedan, Winfried Kockelmann, Anna Fedrigo, Andrei Victor Sandu, Petrica Vizureanu, Jitrin Chaiprapa, Dumitru Doru Burduhos Nergis

**Affiliations:** 1 Faculty of Chemical Engineering Technology, Universiti Malaysia Perlis (UniMAP), Perlis 02600, Malaysia; izrulizwan@unimap.edu.my (M.I.I.R.); mustafa_albakri@unimap.edu.my (M.M.A.B.A.); ikmalhakem@unimap.edu.my (I.H.A.); 2 Geopolymer & Green Technology, Center of Excellence (CEGeoGTech), Universiti Malaysia Perlis (UniMAP), Perlis 02600, Malaysia; chiying95@outlook.com (T.C.Y.); fifinatasha@unimap.edu.my (N.F.S.); 3 STFC, Rutherford Appleton Laboratory, ISIS Facility, Harwell OX11 0QX, UK; winfried.kockelmann@stfc.ac.uk (W.K.); anna.fedrigo@stfc.ac.uk (A.F.); 4 Faculty of Materials Science and Engineering, Gheorghe Asachi Technical University of Iasi, D. Mangeron 41, 700050 Iasi, Romania; 5 Romanian Inventors Forum, St. P. Movila 3, 700089 Iasi, Romania; 6 National Institute for Research and Development in Environmental Protection INCDPM, Splaiul Independentei 294, 060031 Bucuresti, Romania; 7 Technical Sciences Academy of Romania, Dacia Blvd 26, 030167 Bucharest, Romania; 8 Synchrotron Light Research Institute, Muang, Nakhon Ratchasima 30000, Thailand; jitrin@slri.or.th

**Keywords:** geopolymer, pore, tomography imaging, sintering

## Abstract

Geopolymer materials are used as construction materials due to their lower carbon dioxide (CO_2_) emissions compared with conventional cementitious materials. An example of a geopolymer material is alkali-activated kaolin, which is a viable alternative for producing high-strength ceramics. Producing high-performing kaolin ceramics using the conventional method requires a high processing temperature (over 1200 °C). However, properties such as pore size and distribution are affected at high sintering temperatures. Therefore, knowledge regarding the sintering process and related pore structures on alkali-activated kaolin geopolymer ceramic is crucial for optimizing the properties of the aforementioned materials. Pore size was analyzed using neutron tomography, while pore distribution was observed using synchrotron micro-XRF. This study elucidated the pore structure of alkali-activated kaolin at various sintering temperatures. The experiments showed the presence of open pores and closed pores in alkali-activated kaolin geopolymer ceramic samples. The distributions of the main elements within the geopolymer ceramic edifice were found with Si and Al maps, allowing for the identification of the kaolin geopolymer. The results also confirmed that increasing the sintering temperature to 1100 °C resulted in the alkali-activated kaolin geopolymer ceramic samples having large pores, with an average size of ~80 µm^3^ and a layered porosity distribution.

## 1. Introduction

Geopolymer is an inorganic compound material used in construction as a sealant and heat-resistant material [1]. It is a three-dimensional (3D) aluminosilicate structure that is activated using suitable precursor raw materials. Kaolin is an inorganic material that has been identified as geopolymer-compatible with excellent performance. Wang et al. [2] reported that the kaolin structure is significantly influenced by the calcination temperature. A change in the aluminium species influences the structural changes of geopolymer after being heated to 900 °C. The calcium aluminosilicate framework fills the pores between akermanite crystals after being heated up to 1200 °C.

Apart from the geopolymerization component, sintering plays a vital role in producing geopolymer ceramic. Sintering is defined as a thermally activated adhesion process, which increases the contact between particles and their respective coalescence. Sintering closes some of the open pores, decreasing the water absorption rate and increasing pore strength. The dense heated geopolymer has a glassy phase, making it a ceramic. Traditionally, ceramic vitrification begins at 900 °C, marked by the melting of several solid phases that bind present solid particles, enhancing bonding strength [3,4]. The solid reaction product usually consists of an open-pore volume fraction that was reported to be ~<1–40% [5]. After the sintering process, gas adsorption–desorption and mercury intrusion porosimetry are standard methods used to investigate pore structures [6]. Pores ranging from 1 µm to 0.5 mm are also commonly investigated using SEM and nitrogen adsorption. However, these measurements suffer from several drawbacks, rendering them unsuitable for observing cementitious materials. Both are destructive and can potentially alter pore structures. Therefore, advanced techniques such as tomography using neutron sources have been explored to understand the sintering process’ effect on the pore structure of kaolin-based geopolymer. It has been demonstrated that neutron tomography imaging is a suitable characterization method for pore structures. An understanding of the pore structure after the sintering process can be applied for tailoring the resulting materials’ properties. Also, it has been established that the nondestructive testing (NDT) of high-resolution 3D tomography is beneficial as it elucidates qualitative and quantitative pore formations [7]. The utilization of this tomography technique to investigate porosity and pore size distribution is advantageous and effective. Moreover, extensive quantitative research has been conducted on the pores of ceramic materials such as alumina ceramic using X-ray computed tomography, per Lo. et al. [8]. Nickerson et al. also studied the porosities formed in ceramics and their permeability using X-ray computed tomography [9].

In this study, tomography imaging with a neutron source was used to elucidate the effect of sintering on the pore structure of kaolin-based geopolymer. Neutron attenuation coefficients resulted in different image contrasts relative to those generated by conventional X-ray tomography, producing high-resolution images suitable for determining correlations between pore size, density, and absorption performance. Correlations were linked to the elemental distribution obtained using micro-X-ray fluorescence at a synchrotron source. This work successfully characterized and investigated the pore structure of kaolin-based geopolymer.

## 2. Experimental Section

### Materials, Sample Preparation, and Characterization

**Figure 1:**
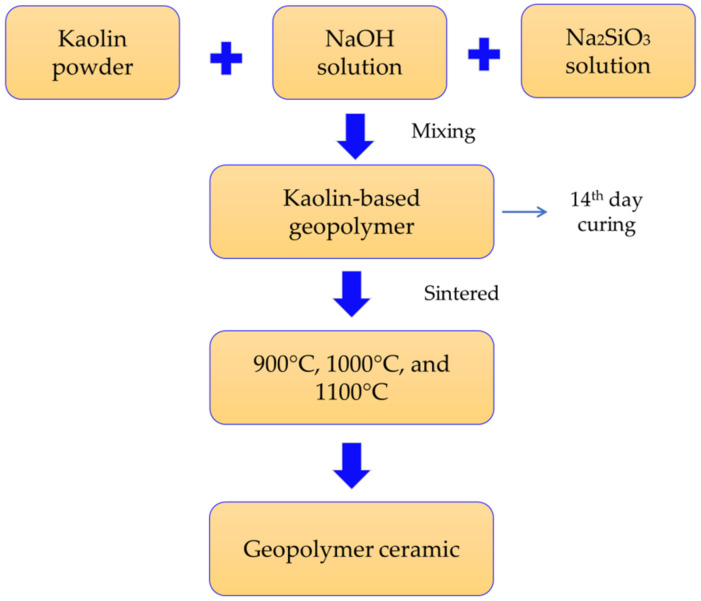
The process of creating kaolin-based geopolymer ceramic.

A precursor of kaolin (supplied by Associated Kaolin Industries Sdn. Bhd., Petaling Jaya, Malasysia) was used for the synthesis of geopolymer. The NaOH was in pellet form with 97% purity, and the Na_2_SiO_3_ consisted of 9.4% Na_2_O, 30.1% SiO_2_, and 60.5% H_2_O, with SiO_2_/Na_2_O = 3.2. The other characteristics were: specific gravity at 20 °C = 1.4 kg/cm^3^ and viscosity = 0.4 Pa s. To form the geopolymer samples, the kaolin was activated with alkaline activator solution, namely, sodium hydroxide (NaOH) and sodium silicate (Na_2_SiO_3_) solution, at ambient temperature. The NaOH clear solution was mixed with sodium silicate solution and cooled to ambient temperature one day before mixing [10]. The solid–liquid and Na_2_SiO_3_/NaOH were fixed at 1.0 (NaOH molarity 8 M) and 1.5, respectively, on the basis of previous research on the optimum design of kaolin geopolymer [11]. The kaolin materials were mixed with an alkaline activator solution for 5 min; then, the homogenized mixture was poured into a mold. Then, after curing for 14 days, the kaolin-based geopolymer was sintered at 900, 1000, and 1100 °C for 2 h at a heating rate of 10 °C/min in an electrically heated furnace. The details of sample preparation are illustrated in Figure 1.

The unsintered and sintered samples of pore microstructures were imaged using the JSM-6460LA Scanning Electron Microscope (JEOL, Peabody, MA, USA) equipped with secondary electron detectors. The voltage and working distance were fixed at 10 kV and 10 mm, respectively. The surface area and pore volume were measured using Brunauer–Emmet–Teller (BET) equipment (TriStar 3000, Micromeritics Instrument Corporation, GA, USA). The adsorbed quantity correlated with the particles’ total surface areas and pore volume in the unsintered and sintered samples. The samples’ thicknesses were 0.5–1 mm. For contrast variation measurements, the samples were placed horizontally in a sample holder and the solvent was added dropwise to the center of the disc.

Neutron images of the samples were acquired at the IMAT beamline, ISIS neutron source, Rutherford Appleton Laboratory, United Kingdom [12]. The IMAT tomography camera was equipped with a 2048 × 2048 pixel Andor Zyla sCMOS 4.2 PLUS. The camera pixel size was 29 µm. The samples were inserted into an aluminum tube that was fixed on the rotating platform and placed at a distance, L, of 10 m from the beam aperture and a distance, d, of 25 mm from the neutron screen. The diameter (D) of the beam aperture was 40 mm, resulting in an L/D ratio of 250. We collected 868 projections, with an exposure time for each projection of 30 s and a total scan time of approximately 6 h/tomogram. Several open-beam and dark images were collected for flat fielding before and after each tomography scan. The images were analyzed using ImageJ and the Octopus reconstruction package (XRE, Ghent, Belgium). The unsintered and sintered geopolymer samples’ elemental distributions were determined using synchrotron µ-XRF at BL6b beamline at the Synchrotron Light Research Institute (SLRI) in Bangkok, Thailand. A polycapillary lens was used to initiate a micro-X-ray beam with a size of 30 × 30 µm on the samples, with continuous synchrotron radiation. The X-ray energy range used was 2–12 keV without the monochromator feature. The detection limit at the sub parts per million concentration level can be obtained at larger than 100 nm, with sensitivities approaching the attogram (10–18 g) level [13]. The experiments were conducted in a helium (He) gas atmosphere with 30 s of exposure at each point. The data were obtained and analyzed using PyMca [14].

The samples were fabricated in powder form for phase analyses. The XRD analysis was performed using an XRD-6000 Shimadzu X-ray diffractometer (Cu Kα radiation (λ = 1.5418 A)). The operating parameters were 40 kV, 35 mA, at 2θ of 10–80°, at a 1°/min scan rate. The XRD patterns were then analyzed using X’pert HighScore Plus. The density was calculated, and water absorption tests were conducted per ASTM C642-13 (ASTM C642-13, Standard Test Method for Density, Absorption, and Voids in Hardened Concrete, ASTM International, United States (2013)). The weight of the samples after and before the samples were immersed in water was recorded, and the percentages of water absorption for the samples after sintering at 900 and 1100 °C were determined.

## 3. Results and Discussions

### 3.1. Density and Water Absorption Analysis

**Figure 2:**
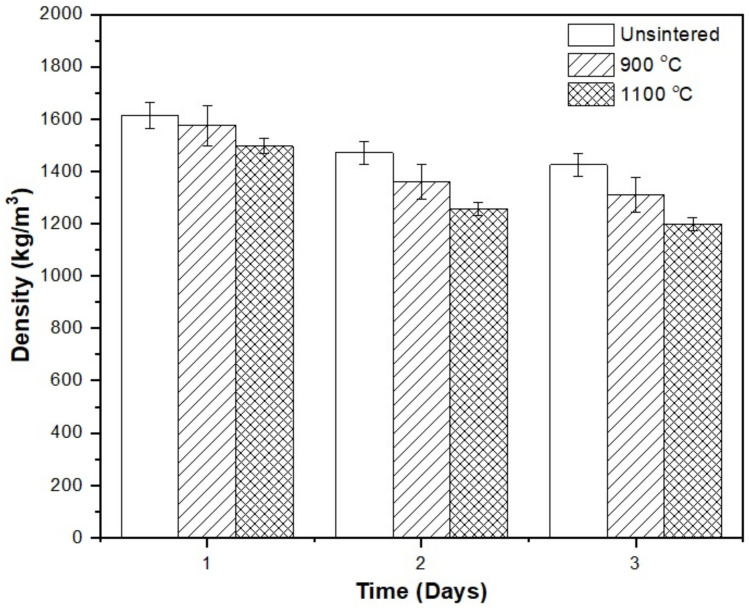
The density of kaolin geopolymer over 3 days for unsintered and sintered samples at 900 and 1100 °C.

In order to examine the pore structure in kaolin-based geopolymer ceramic, the density and water absorption of kaolin-based geopolymer samples were investigated. The densities of the unsintered and sintered kaolin at 900 and 1100 °C after 3 days are shown in Figure 2. The densities of the unsintered and sintered samples at all temperatures decreased as time increased. The unsintered samples had the highest density of 1610 kg/cm^3^, while the samples sintered at 1100 °C had the lowest density of 1203 kg/cm^3^. Therefore, we speculate that the formation of large pores created in the kaolin at 1100 °C resulted in the lowest density, while sintering at 900 °C resulted in the formation of small pores in the kaolin-based geopolymer samples. In addition, the unsintered samples contained small and open pores, while the sintered samples had large and closed pores, which translated into a high material density. The existence of these larger pores was likely due to the growth of sintered necks, which was reflected in the phase evolution. The details of phase crystallization are discussed in Section 3.6.

**Figure 3:**
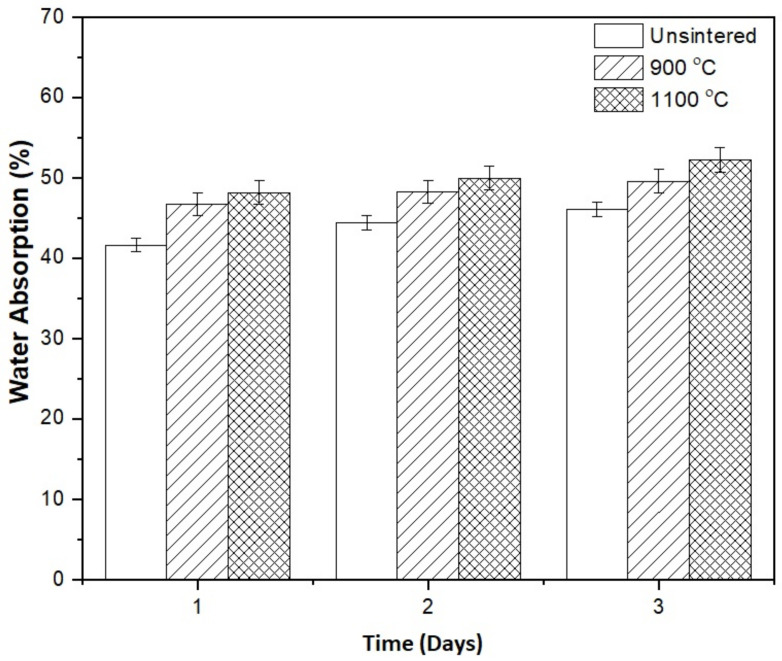
The water absorption of kaolin geopolymer over 3 days for unsintered and sintered samples at 900 and 1100 °C.

Figure 3 shows the percentage water absorption of the kaolin-based geopolymer ceramic samples after sintering at 900 and 1100 °C after 3 days. After 3 days, the highest value percentage water absorption occurred with sintering at 1100 °C. The percentage water absorption continuously increased with sintering temperature and time. This was in accordance with results published by Faris et al. [15]. The higher sintering temperature resulted in larger pores due to water removal, and the increased pore size increased the water absorption capacity of the kaolin-based geopolymer samples. The high volume of open pores in the samples may have contributed to the high water absorption due to a high surface area, which was reported by Zulkifli et al. [11].

### 3.2. Pore Structure Analysis

**Figure 4:**
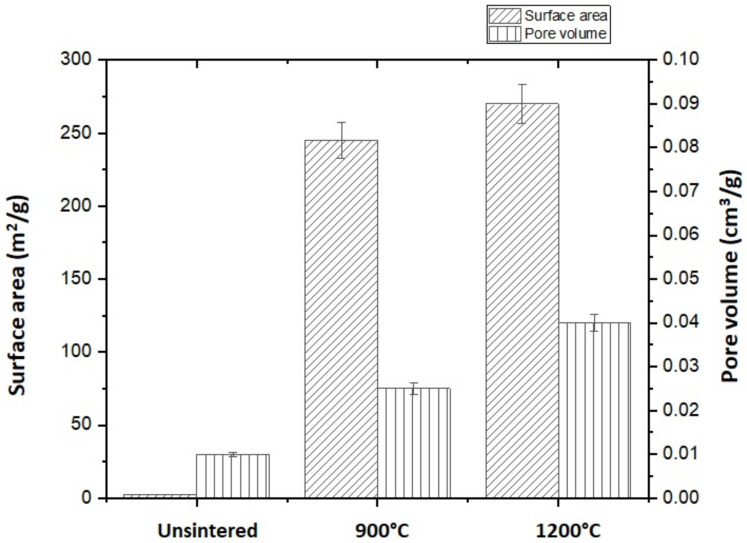
Surface area and pore volume of kaolin geopolymer samples versus sintering temperature.

The Brunauer–Emmett–Teller (BET) method was used to determine the surface area of the unsintered and sintered kaolin-based geopolymers. The specific surface area and pore volume of unsintered and sintered geopolymers are depicted in Figure 4. Smaller particles resulted in larger surface areas. This is because sintered kaolin-based geopolymer has a larger surface area due to the removal of volatiles and impurities from the sample’s surface. Sutama et al. [16] stated that the formation of pores on the sample may lower the compressive strength.

The unsintered kaolin-based geopolymer had the lowest surface area (2.3 m^2^/g) and pore volume (0.01 cm^3^/g). After sintering at 900 °C, the kaolin-based geopolymer’s surface area (up to 245 m^2^/g) and pore volume (up to 0.025 cm^3^/g) increased relative to those of the unsintered kaolin-based geopolymer. Then, after sintering at 1100 °C, the surface area increased to 270 m^2^/g and pore volume increased to 0.04 cm^3^/g. The kaolin-based geopolymer was assumed to consist of mesopores in a small quantity, resulting in a higher surface area after sintering at high temperatures.

### 3.3. Microstructure Analysis

**Figure 5:**
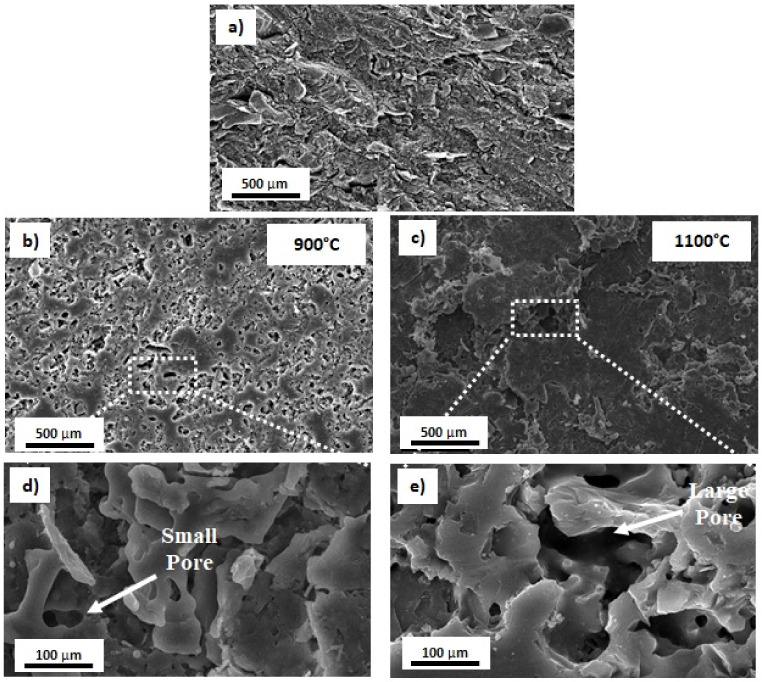
SEM micrograph of (**a**) unsintered, (**b**,**d**) sintered at 900 °C, and (**c**,**e**) sintered at 1100 °C kaolin-based geopolymer.

An SEM revealed the morphological features of kaolin-based geopolymer ceramic samples at sintering temperatures of (a) unsintered, (b) 900, and (c) 1100 °C, as shown in Figure 5. The unsintered kaolin showed the presence of well-defined clay platelets and an incomplete reaction of kaolin, as shown in Figure 5a. After sintering at 900 and 1100 °C, the images clearly showed the presence of pores and cracks in all of the heated kaolin-based geopolymer ceramic samples. The pores formed a network, which resulted in increased internal porosity. The kaolin-based geopolymer surface became glassy and glossy when sintered at 900 °C (Figure 5b). This microstructure change was attributed to moisture hydration and phase transformation, as reported by Dudek et al. [17]. It can also be seen in Figure 5b that the kaolin-based geopolymer ceramic samples sintered at 900 °C had a higher porosity, alongside cracks and voids.

Increasing the sintering temperature up to 1100 °C increased the number of large pores. The pore size distribution of kaolin-based geopolymer was ~50 µm for the unsintered samples. After sintering at 1100 °C, the pore size increased to 80 µm, similar to the findings from the tomography analysis. The pore sizes in kaolin directly affect its mechanical strength. The SEM images also showed significant cracks due to moisture evaporation and shrinkage during the sintering process. The loosely grained structure of kaolinite can also cause cracks, and the presence of voids at the interface of loosening grains can result in increased total porosity.

### 3.4. Neutron Tomography Imaging Analysis

**Figure 6:**
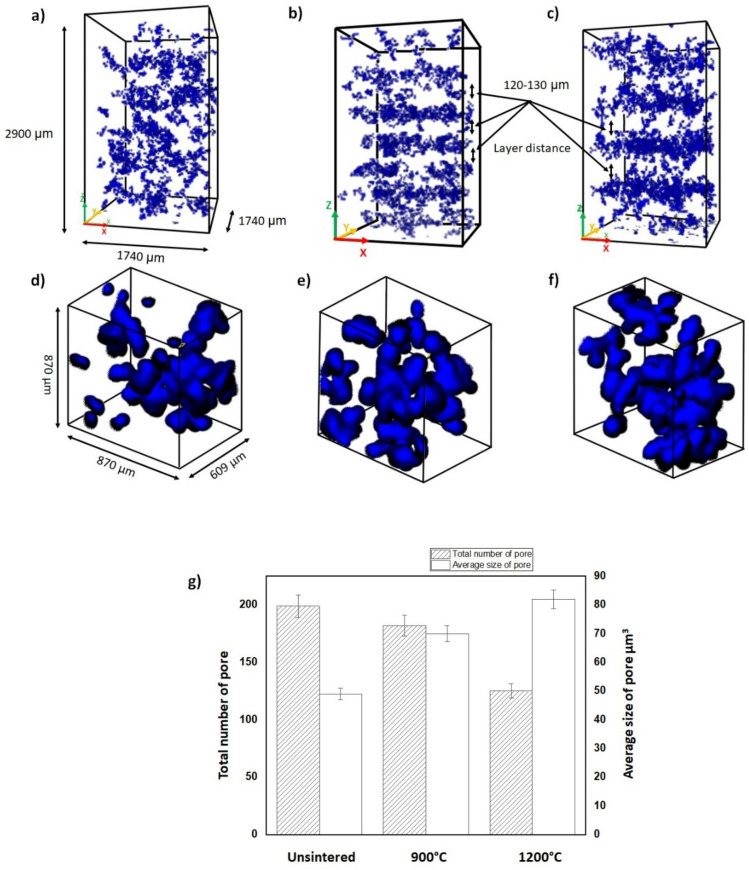
Tomography imaging of (**a**) unsintered and sintered geopolymer at (**b**) 900 and (**c**) 1100 °C. (**d**–**f**) Tomography imaging with zoom and higher resolution and (**g**) total pore numbers and average pore sizes.

Segmentation was carried out in a small area to analyze porosity data in the kaolin-based geopolymer samples quantitatively, and the 3D reconstruction images are shown in Figure 6. The kaolin-based geopolymer samples’ widths, lengths, and thicknesses, shown in Figure 6a–c, were 2900, 1740, and 1740 µm, respectively. The white color indicates the solid kaolin-based geopolymer, while blue indicates the air (pore) space. The total number of pores for this region was estimated to be 197, and after sintering at 900 and 1100 °C, the total number of pores decreased to 182 and 125, respectively. Neutron tomography made imaging very small pores at high resolutions possible, and the results are shown in Figure 6d–f. In the case of the unsintered kaolin, the pore size was ~50 µm^3^, and when sintered at 900 and 1100 °C, the pore size increased to 68 and 82 µm^3^, respectively. Figure 6g displays pore numbers and sizes. These sizes are in agreement with those measured in the SEM images shown in Figure 4.

When sintered, the small pores merged to become large(r) pores due to moisture hydration after sintering. Our images show the isolated closed pores in the 3D volume, and it was, in fact, a network of fully connected open pores in 3D. Interestingly, after sintering, the pore distribution of the kaolin-based geopolymer became layered, as shown in Figure 6b,c. The layer distance between porosities was estimated to be ~120-130 µm when sintered at 900 and 1100 °C because the kaolin-based geopolymer exhibited low reactivity with the alkaline silicate solution.

**Figure 7:**
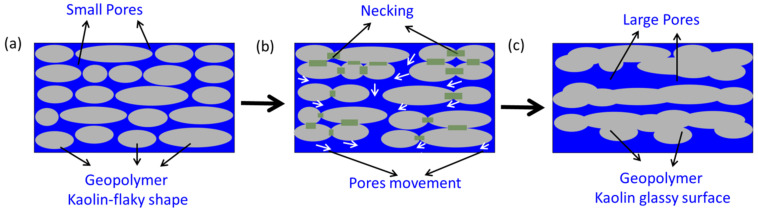
Sintering mechanism of pore transformation in various environments: (**a**) unsintered, (**b**) 900 °C, and (**c**) 1100 °C.

A layered structure was caused by the sintering of the kaolin-based geopolymer at a higher temperature. The layered structure was indicated by the transformation of pore appearance, as shown in Figure 7. The pore transformation was attributed to the larger surface area causing necking reactions between particles (Figure 7b). During sintering, atoms diffuse from an area of higher chemical potential to an area of lower chemical potential. Small pores then merge to form larger pores. The layered grain structure represented the disorganized kaolinite structure (grey color) that was due to dehydroxylation. The dehydroxylation of kaolin resulted in the destruction of the crystalline structure and the transformation of the mullite phase, as confirmed by an XRD analysis. These findings are consistent with ElDeeb et al. [18], who posited that the hydroxylation of clay sheets occurs with high-temperature sintering.

### 3.5. Elemental Distribution Analysis

**Figure 8:**
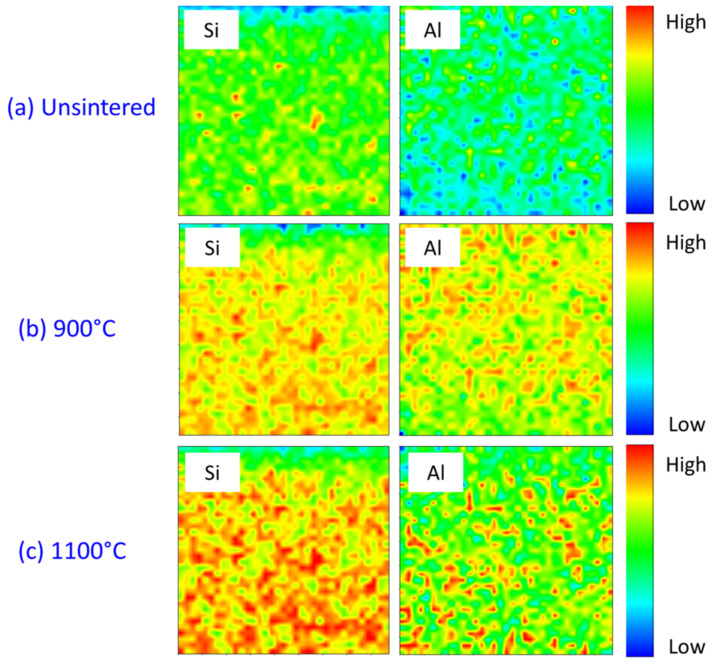
Micro-XRF elemental distribution maps of Si and Al in kaolin geopolymer ceramic at various sintering temperatures.

The kaolin-based geopolymer ceramic samples were further characterized using micro-XRF mapping to understand their elemental distribution vis-à-vis sintering temperatures. Figure 8 illustrates the localized micro-XRF mapping of the kaolin-based geopolymer ceramic samples that were (Figure 8a) unsintered or heated to 900 (Figure 8b) or 1100 °C (Figure 8c), signifying where the (main) elements Si and Al were critically located within the samples. The distributions of the main elements within the geopolymer ceramic edifice were confirmed using synchrotron micro-XRF. The distribution of Si combined with the Al map allowed for the identification of the kaolin-based geopolymer ceramic backbone (kaolinite). The red, green, and blue spots represent the high, medium, and low intensities, respectively, for each distribution element at the integrated area.

The various sintering temperatures resulted in significant changes in the Si and Al element distributions, edging the material towards phase transformation. A high concentration of Si (Figure 8a) represented the kaolinite grain. Upon obtaining the pore microstructure of the kaolin-based geopolymer ceramic (Figure 8b), the Si and Al regions showed higher intensities, reflecting the presence of the minerals quartz and nepheline, as depicted in Figure 8 and the next section. At 1100 °C, Si and Al were of higher intensities in a localized area, reflecting the formation of mullite. This Si–Al-rich crystalline mineral contributed to the pores’ microstructure appearance, as shown in Figure 5b,c.

### 3.6. Mineral Phase Transformation

**Figure 9:**
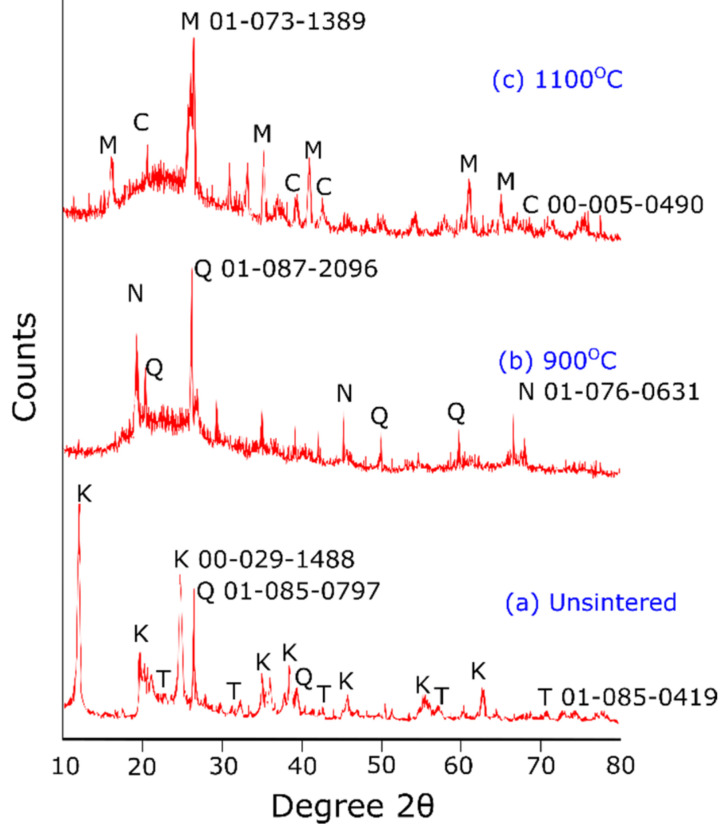
Phase transformation of kaolin geopolymer when (**a**) unsintered, (**b**) sintered at 900 °C, and (**c**) sintered at 1100 °C. M, mullite; C, cristobalite; Q, quartz; K, kaolin; N, nepheline; T, tridymite.

Figure 9 shows an XRD diffractogram of the kaolin-based geopolymer ceramic when (a) unsintered or heated to (b) 900 or (c) 1100 °C. The unsintered kaolin-based geopolymer showed the presence of crystalline phases such as kaolinite, quartz, and tridymite. A geopolymerization reaction was initiated by the dissolution of aluminosilicate materials in an alkali activator (combination of NaOH and Na_2_SiO_3_ solutions). Next, the products of dissolution underwent nucleation growth and polymerization processes before hardening at the polycondensation stage. There have been several findings obtained with a similar method for producing kaolin-based geopolymer at room temperatures [19,20]. Additionally, kaolinite was traced as a major mineral in spectra of kaolin-based geopolymer samples [21]. Owing to the lower activity of pure kaolin, a number of distinctive kaolinite peaks remained in the diffractogram of the kaolin-based geopolymer [22]. However, these kaolinite peaks decreased at high sintering temperatures, as shown in Figure 9b.

Sintering temperatures up to 1100 °C introduced the formation of the mullite phase (Figure 8c). The mullite phase is present in this sintering region, manifesting superior thermochemical stabilities [23,24]. Furthermore, the appearance of cristobalite was due to unreacted quartz (SiO_2_) after the decomposition of kaolinite at 900 °C [25]. The liberation of SiO_2_ corresponded to the kaolinite–mullite transformation, which yields to Al–Si spinel phase [26]. This was corroborated with the elemental distribution analysis obtained using micro-XRF, as the sintered kaolin geopolymer ceramic samples showed a high intensity at the Si and Al regions at 1100 °C (Figure 7c). The transformation of mullite is described by the chemical reaction in Equation (1) [27,28]:

2Si_3_Al_4_O_12_Al-Si spinel → 23Al_2_O_3_.2SiO_2_ mullite + 5SiO_2_ (1)

## 4. Conclusions

This manuscript summarizes the effects of sintering temperature on the pore structure of an alkali-activated kaolin-based geopolymer ceramic. Sintering temperatures significantly affected the size and number of pores in the kaolin-based geopolymer. The material’s density and water absorption confirmed the presence of pores after the sintering process. Microstructural analyses showed that sintering at 1100 °C resulted in large pore sizes relative to the material’s unsintered counterpart. Tomography imaging also confirmed the presence of a layered pore structure after sintering. The pore size at 900 °C was 50 µm^3^, and after sintering at 900 and 1100 °C, the pore size increased to 68 and 82 µm^3^, respectively.

## Data Availability

Not applicable.
